# Comorbid Mental Disorders and 6-Month Symptomatic and Functioning Outcomes in Chinese University Students at Clinical High Risk for Psychosis

**DOI:** 10.3389/fpsyt.2017.00209

**Published:** 2017-10-23

**Authors:** Jingyu Shi, Lu Wang, Yuhong Yao, Na Su, Chenyu Zhan, Ziyu Mao, Xudong Zhao

**Affiliations:** ^1^East Hospital, Tongji University School of Medicine, Shanghai, China; ^2^Tongji University School of Medicine, Shanghai, China; ^3^Students Counseling Center, Tongji University, Shanghai, China

**Keywords:** clinical high risk, psychosis, depression, anxiety, university students, longitudinal

## Abstract

**Background:**

High rates of non-psychotic psychopathological symptoms have been observed in clinical population at clinical high risk (CHR) for psychosis. These comorbid disorders affected the baseline functional level of CHR patients. However, little is known about the comorbid mental disorder in CHR individuals in non-clinical adolescent population. This study aimed to investigate the comorbid mental disorder in non-clinical CHR adolescents and the impact on attenuated psychosis symptoms (APS) as well as clinical outcome.

**Methods:**

The sample consisted of 32 CHR students, who were screened from 2,800 university students. CHR status was evaluated with the Structured Interview of Prodromal Syndromes, comorbid mental disorder diagnoses with the International Neuropsychiatric Interview.

**Results:**

In the CHR sample, 46.9% was found at least one non-psychotic comorbid mental disorder. The CHR participants presenting comorbid mental disorder had significantly more severity of APS than those without comorbid mental disorders, and the remission rate at 6-month follow-up is significantly higher in the individuals without comorbid mental disorders at baseline.

**Conclusion:**

In the non-clinical sample of individuals at CHR, non-psychotic comorbid mental disorders are common and anxiety disorder is most frequent. Copresence of anxiety and/or depression is related to higher level of attenuated psychotic symptoms and unfavorable clinical outcome at 6-month follow-up. Assessment and intervention in anxiety and depression for non-clinical CHR adolescents are suggested.

## Introduction

Psychoses typically emerge in late adolescence or early adulthood, which are among the main causes of disability in the general population (1). Approximately 20% of young people experience a mental health problem every year (2, 3). Indeed, epidemiological research indicates that the majority of individuals first experience mental health symptoms before the age of 24 years (4). University students are in late adolescence and early adulthood; this stage belongs to the high-incidence age for psychosis. The emergence of serious mental health problems can disrupt social and psychological development, including the attainment of educational goals and relationship skills, thus seriously impairing their quality of life. In addition, researchers have suggested that students with mental health problems face high risk of dropping out of college (5). Psychosis has been described as a serious mental illness due to the associated disability across the life span including lost opportunity for education, employment, and relationships (6).

The criteria for being at risk for psychosis have been developed to prospectively identify people experiencing a potential prodrome stage for this illness (7, 8). These criteria are mainly based on attenuated psychotic symptoms (APS), and the term clinical high risk (CHR) is applied to describe this population (7, 8). The CHR criteria provide an important opportunity for early detection and intervention in preventing or postpone the onset of psychosis in reducing the social and economic burden associated with long-term mental health problems. In addition, the CHR state often copresents with other non-psychotic mental disorders (9, 10). In the previous study, it was found that in CHR patients, approximately 73% of them indicated at least one coexisting Axis I diagnoses in addition to the CHR state––the most common being of depression and/or anxiety disorders (11). The cooccurrence of the mental disorders impacts the functional level of CHR patients at baseline, with a cumulative effect of the comorbid anxiety and depressive disorders (11). These results suggest that CHR patients suffer from painful emotions and disability in their own mental difficulties, regardless of the development of psychotic disorders. In fact, the presence of psychopathological symptoms in addition to APS is the most common complaint that triggers help-seeking behaviors among this population (12, 13). However, studies on CHR state have mostly focused on the help-seeking patients without venturing into the non-clinical population. In particular, research on the CHR state in samples of university students is not fully explored. Until now, little is known about the non-psychotic psychopathological symptoms in CHR university students and the impact on psychopathology and clinical outcome in this population. Therefore, the present study aimed to (1) investigate the comorbid mental disorder in CHR individuals among non-clinical adolescents and (2) explore the association of the comorbid mental disorder with psychotic symptomatic and general functioning outcome, in order to establish the early detection and prevention system in non-clinical population.

## Materials and Methods

### Participants

In this study, the participants were 32 university students at CHR state, including 13 male and 19 female aged from 17 to 21 years, with an average of 18.78. The 32 subjects were screened from 2,800 university students of the first and second grades. The status of CHR was diagnosed through a two-stage assessment, including screening the potential subjects with questionnaire and assessment by Structured Interview for Psychosis-Risk Syndromes (SIPS) (14). This study had ethics approval from the institutional review board of Tongji University. Prior to the study each participant provided written informed consent. For the participants under 18 years old, we have contacted with their parents and obtained informed consent from them.

### Assessments

#### Instruments for Detection

The 16-item Prodromal Questionnaire (PQ-16) is a self-report screening tool for individuals at risk of psychosis. It includes nine items concerning hallucinations and perceptual abnormalities, five items on symptoms as delusional thoughts, paranoia and unusual thinking content, and the other two items about negative symptoms (15). According to the subjective experience of the participants in the last month, the items were marked as yes or no. If an item was selected as “no,” this item was rated 0; when an item was selected as “yes,” the severity of the distressing experience ranging from 0 (none) to 3 (severe) was further rated. The final score was the total score of each item. The PQ-16 was applied in Chinese population for screening individuals at risk of psychosis and demonstrated good reliability and validity (16). In this study, the internal consistency reliability of PQ-16 was good (Cronbach’s α = 0.72).

The SIPS is a semi-structured interview designed to assess the risk of psychosis, which is conducted by clinician. The SIPS assessed the severity of symptoms in four areas (positive, negative, disorganized, and general symptoms) on a rating scale ranging from 0 (representing the absence of a symptom) to 6 (representing “severe and psychotic”). If the severity of a positive symptom is rated within the range of 3–5, that symptom is considered as an “attenuated psychotic symptom.” There are three types of Psychosis-Risk Syndromes diagnosed in SIPS: (1) Attenuated Positive Symptom Prodromal Syndrome (APSS), defined as attenuated positive psychotic symptoms recently occurring with sufficient frequency or severity; (2) Brief Intermittent Psychosis Prodromal Syndrome (BIPS), defined as psychiatric symptoms with spontaneous remission presenting within 1 week; and (3) Genetic Risk and Deterioration Prodromal Syndrome (GRDS), defined as both of genetic risk and recent functional decline existing. The reliability and validity of this Chinese version is demonstrated good in the assessment of psychosis risk syndromes (17). The internal consistency reliability of the SIPS in this study was good (Cronbach’s α = 0.80).

#### Instruments for Symptoms

The severity of prodromal symptoms was used as measure of the outcome, which was rated by the Scale of Prodromal Symptoms in the SIPS. The overall functioning was assessed with the Global Assessment of Functioning (GAF) scale (18). The Mini International Neuropsychiatric Interview was used to assess the comorbid mental disorders. The Mini International Neuropsychiatric Interview is a brief structured interview for the major Axis I mental disorders in the Diagnostic and Statistical Manual of Mental Disorders, fourth edition (DSM-IV) and International Classification of Diseases, 10th edition (ICD-10) (19).

#### Procedure

In the first stage, the PQ-16 was distributed to 2,800 university students of the first and second grades, in order to screen out the potential population at CHR for psychosis. The questionnaire was given to the students at the end of the mental health education course. They volunteered to participant in the survey and they were instructed to complete the PQ-16 and social-demographic information in a quiet classroom environment after informing consent. It took approximately 5 min for the students to finish it. Among them 2,560 students completed the investigation, and out of these 224 (8%) responds were invalid and were not included in the analysis. The effective response rate is 83.4%. There were no statistical difference between the basic population (2,800 students) and the responders by gender and age (*p* > 0.05). The positive threshold of this tool was 6, according to the study carried out by Ising et al. (15). In total, 611 students reached this cutoff score, and we obtained agreement from 529 participants who accepted SIPS. There were no statistical difference between the SIPS-evaluated students (*n* = 529) and the screen positive students (*n* = 611) by gender and age (*p* > 0.05). The SIPS was conducted for them by trained psychiatrists. Thirty-two of them met the criteria of CHR on SIPS. The inclusion and exclusion criteria for CHR individuals are listed in Table 1. Mini International Neuropsychiatric Interview was conducted by psychiatrists for the 32 CHR individuals. Among the 32 CHR subjects, 27 participants completed the assessment in 6-month follow-up. Out of the 32 CHR individuals, three students cannot be reached with the original phone number and two students refused to participate it anymore. The SIPS were conducted for the 27 subjects by the same psychiatrists at 6-month follow-up.

**Table 1:** Inclusion and exclusion criteria for CHR.

Inclusion criteria	Exclusion criteria
Age between 16 and 30 years Presence of at least one of the following criteria based on SIPS (1) APSS (2) BIPS (3) GRDS	Any history of the following disorders: Schizophrenia, schizophreniform disorder, schizoaffective disorder, delusional disorder, bipolar disorder. Somatic disorder-induced mental disorder, psychoactive substance-induced mental disorder. Substance abuse Organic brain damage, epilepsy Continuously taking antipsychotic drugs, antidepressants and emotional stabilizers for more than 2 weeks over the past 3 months

*APSS, Attenuated Positive Symptom Prodromal Syndrome; BIPS, Brief Intermittent Psychosis Prodromal Syndrome; CHR, clinical high risk; GRDS, Genetic Risk and Deterioration Prodromal Syndrome; SIPS, Structured Interview for Psychosis-Risk Syndromes*.

### Statistical Analyses

The statistical analysis was carried out using the Statistical Package for the Social Sciences (SPSS 17.0). *T*-tests for independent samples were used to assess differences in SIPS psychopathologies score between the two groups with and without comorbid mental disorders. Spearman’s correlations were conducted to assess how comorbid mental disorders were related with the severity of APS and GAF. Chi-square tests were employed to compare the remission rate between the two groups with and without comorbid mental disorders at baseline.

## Results

### Sociodemographic and Baseline-Attenuated Psychotic Characteristics

The 32 CHR individuals aged from 17 to 21 years volunteered to participate in this study, with an average of 18.78. All of them were unmarried. Among the 32 subjects, 19 of them were females. All the detected subjects at CHR state were diagnosed as APSS, and seven participants also met the criteria for GRDS. The most common APS (severity score ≥ 3) were Perceptual Abnormalities/Hallucinations (87.5%), Unusual Thought Content/Delusional Ideas (56.3%), and Suspiciousness/Persecutory Ideas (18.8%) in positive symptoms. The GAF score of the CHR subjects was 71.53 ± 7.39, and this level suggested slight impairment in social or school functioning. Detailed results of SIPS individual items are provided in Table 2.

**Table 2:** Baseline-attenuated psychotic characteristics.

Item		*N*^a^ (%)	Mean ± SD	Median (Q1, Q3)
Positive symptoms		32 (100.0)	7.81 ± 3.542	7.00 (4.25, 10.00)
P1	Unusual thought content/delusional ideas	18 (56.3)	2.38 ± 1.621	3.00 (1.00, 4.00)
P2	Suspiciousness/persecutory ideas	6 (18.8)	1.34 ± 1.558	1.00 (0.00, 2.00)
P3	Grandiose ideas	2 (6.3)	0.50 ± 0.950	0.00 (0.00, 1.00)
P4	Perceptual abnormalities/hallucinations	28 (87.5)	2.88 ± 1.040	3.00 (3.00, 3.00)
P5	Disorganized communication	1 (3.1)	0.72 ± 0.991	0.00 (0.00, 1.00)
Negative symptoms		10 (31.3)	5.16 ± 5.100	4.00 (1.00, 8.75)
N1	Social anhedonia	6 (18.8)	1.34 ± 1.473	1.00 (0.00, 2.00)
N2	Avolition	3 (9.4)	0.88 ± 1.212	0.00 (0.00, 1.75)
N3	Expression of emotion	5 (15.6)	0.84 ± 1.221	0.00 (0.00, 1.75)
N4	Experience of emotions and self	5 (15.6)	1.00 ± 1.136	1.00 (0.00, 2.00)
N5	Ideational richness	0 (0.0)	0.28 ± 0.634	0.00 (0.00, 0.00)
N6	Occupational functioning	1 (3.1)	0.81 ± 0.965	0.00 (0.00, 1.00)
Disorganized symptoms		7 (21.9)	2.97 ± 2.935	2.00 (0.25, 4.75)
D1	Odd behavior of appearance	1 (3.1)	0.50 ± 0.984	0.00 (0.00, 0.75)
D2	Bizarre thinking	7 (21.9)	1.09 ± 1.254	0.50 (0.00, 2.00)
D3	Trouble with focus and attention	3 (9.4)	1.31 ± 1.091	1.50 (0.00, 2.00)
D4	Impairment in personal hygiene	0 (0.0)	0.00 ± 0.246	0.00 (0.00,0.00)
General symptoms		8 (25.0)	3.50 ± 2.828	3.50 (1.00, 5.75)
G1	Sleep disturbance	4 (12.5)	0.84 ± 1.221	0.00 (0.00, 2.00)
G2	Dysphoric mood	5 (15.6)	1.25 ± 1.244	1.00 (0.00, 2.00)
G3	Motor disturbances	0 (0.0)	0.47 ± 0.671	0.00 (0.00, 1.00)
G4	Impaired tolerance to normal stress	1 (3.1)	0.94 ± 0.914	1.00 (0.00, 2.00)
GAF			71.53 ± 7.392	73.50 (68.50, 78.00)

*^a^Present if score ≥3*.

*GAF, Global Assessment of Functioning*.

### Comorbid Mental Disorders in Subjects at CHR for Psychosis at Baseline

Mini International Neuropsychiatric Interview was conducted by psychiatrists for the 32 CHR individuals. Fifteen (46.9%) subjects were found at least one comorbid psychopathological symptoms. Among them, nine subjects (28.1%) had anxiety disorder and six subjects had both of anxiety and depression disorders (18.8%). The most common three comorbid mental disorders were generalized anxiety disorder, social phobia, and obsessive-compulsive disorder. The participants were not treated for their comorbid mental disorders. The distribution of comorbid psychopathology is presented in Table 3.

**Table 3:** Distribution of comorbid psychopathology in CHR individuals.

Comorbid psychopathology	*n* (%)
Generalized anxiety disorder	8 (25)
Social phobia	6 (18.8)
Obsessive-compulsive Disorder	4 (12.5)
Panic attack	3 (9.4)
Agoraphobia	3 (9.4)
Specific phobia	3 (9.4)
Depression	3 (9.4)
Dysthymia	3 (9.4)
Somatization disorder	1 (3.1)
Body dysmorphic disorder	1 (3.1)

*CHR, clinical high risk*.

### Comparison of Severity of Psychotic Symptoms and Level of Functioning between Groups at Baseline

Significant differences were observed in severity of APS in groups with and without comorbid psychopathological symptoms (*p* < 0.05) at baseline. The mean ± SD, *t*-value, and *p*-value are provided in Table 4.

**Table 4:** Severity of attenuated psychotic symptoms and GAF in two groups (*x* ± SD).

	Group with CPS (*n* = 15)	Group without CPS (*n* = 17)	*t*	*p*
PS	9.13 ± 2.924	6.65 ± 3.707	−2.086	0.046
NS	7.13 ± 5.423	3.41 ± 4.214	−2.181	0.037
DS	3.67 ± 3.155	2.35 ± 2.668	−1.276	0.212
GS	4.60 ± 2.823	2.53 ± 2.528	−2.189	0.036
GAF	70.47 ± 8.935	72.47 ± 5.832	0.760	0.453

*CPS, comorbid psychopathological symptoms; DS, disorganized symptoms; GAF, Global Assessment of Functioning; GS, general symptoms; PS, positive symptoms; NS, negative symptoms*.

### Association between Baseline Comorbid Mental Disorders and Baseline Severity of Attenuated Psychotic Symptoms and Global Functioning

The presence of comorbid disorders (“1” for “no,” “2” for “yes”) had a significant association with baseline SIPS psychopathology in the current sample. There was a significant positive correlation with the SIPS subscales addressing Suspiciousness/Persecutory Ideas (*r* = 0.578, *p* = 0.001), Social Anhedonia (*r* = 0.600, *p* = 0.000), Avolition (*r* = 0.456, *p* = 0.009), Dysphoric Mood (*r* = 0.463, *p* = 0.088), and Impaired Tolerance to Normal Stress (*r* = 0.378, *p* = 0.033); lower SIPS scores in subjects with no comorbid disorders and higher SIPS scores in subjects with comorbid disorders.

### Association between Baseline Comorbid Mental Disorders and 6-Month Symptomatic and Global Functioning Outcome

Among the 32 CHR subjects, 27 participants completed the assessment in 6-month follow-up. Among the 27 subjects, 14 individuals had no comorbid mental disorders at baseline and the other 13 individuals were with comorbid mental disorders at baseline. In the group without comorbid mental disorders, 11 (78.6%) CHR individuals had fully remission from an initial CHR status and 3 (21.4%) CHR individuals still accord with the CHR criteria. However, in the group with comorbid mental disorders, a psychotic manic episode of bipolar I disorder was observed in one subject during the follow-up period. The conversion rate at 6-month follow-up was 3.7% (1/27), eight (61.5%) CHR individuals still met CHR criteria, and the other four (30.8%) had a fully remission. The remission rate is significantly higher in the individuals without comorbid mental disorders at baseline (χ^2^ = 6.238, *p* = 0.013). In addition, the correlation analysis between the presence of comorbid disorders at baseline and the SIPS symptoms as well as functioning outcomes 6 months later was conducted. The results showed that the presence of comorbid disorders at baseline was positively correlated with (1) the scores of subscales of positive symptoms addressing Unusual Thought Content/Delusional Ideas (*r* = 0.411, *p* = 0.041), Suspiciousness/Persecutory Ideas (*r* = 0.460, *p* = 0.000), and Disorganized Communication (*r* = 0.422, *p* = 0.036); (2) the scores of subscales of negative symptoms addressing Avolition (*r* = 0.423, *p* = 0.035); and (3) the scores of subscales of general symptoms addressing Dysphoric Mood (*r* = 0.457, *p* = 0.022).

## Discussion

The current study investigated the comorbid non-psychotic mental disorder in CHR individuals among university students, and explored the association of the comorbid mental disorder with psychotic symptoms and functioning outcome in this population at 6-month follow-up. About half of the baseline sample (46.9%) was found at least one non-psychotic comorbid mental disorder. Among those who presented non-psychotic comorbid mental disorders showed more serious APS as compared with those without comorbid mental disorders. Moreover, the remission rate at 6-month follow-up is significantly higher in the individuals without comorbid mental disorders at baseline.

The first aim of this study was to assess the prevalence of non-psychotic comorbid mental disorders in non-clinical adolescent population at CHR for psychosis. In the 32 CHR subjects, who were screened from 2,800 students, we found a high prevalence (46.9%) of non-psychotic comorbid mental disorder in addition to the at-risk signs and symptoms. In total, 37.5% of the CHR students presented one or more anxiety disorders, with general anxiety disorder being the most common (25%), while 18.75% of the students had a comorbid diagnosis of depressive disorders. Our results are in accordance with previous studies suggesting that a significant proportion of CHR individuals occur with non-psychotic mental disorders, and the most common diagnoses are anxiety and/or depression (9, 20–22), which are frequently confirmed as the primary subjective complaints that trigger CHR individuals to seek help (13). Anxiety may be particularly prominent in the non-help-seeking CHR population, and it was reported that among the people at CHR state, 32% meet criteria for an anxiety disorder (23). However, a meta-analysis about the clinical samples found that the baseline comorbid anxiety and depressive disorders presented in 8 and 40% of subjects, respectively (11). It seems that in the non-help-seeking CHR subjects, anxiety disorders are common, while in the help-seeking CHR subjects depressive disorders are prominent. Several potential reasons could be suggested to explain the frequent presence of anxiety in CHR youth in non-clinical population. On one hand, it was postulated that vulnerability to various psychiatric symptoms may exist in a continuum, namely, that the one who is vulnerable to either depression or anxiety is also vulnerable to develop APS and *vice versa* (24). On the other hand, it has been hypothesized that the cooccurrence of APS and anxiety may not mean representation of two different disorders, but they could be a single disorder with diverse symptoms (11). However, the cooccurring depressive symptoms might promote individuals to seek help (25), which resulted in a high percentage of prevalence in depression in CHR subjects in clinical samples, while anxiety symptoms may be not the first triggers that make the CHR individuals seek help. Furthermore, multiple other differences could exist between help-seeking and non-help-seeking individuals with prodromal syndromes. Further longitudinal and cross-sectional study on comparing clinical and community samples will be necessary to explore the characteristics of comorbid mental disorders in CHR population.

The second aim of the study was to investigate the association between baseline comorbid mental disorder and short-term APS and general functioning outcomes. In the current study at 6-month follow-up, the remission rate is significantly higher in the individuals without comorbid mental disorders at baseline; one CHR individual with depression disorder and generalized anxiety disorder at baseline developed a psychotic episode during this follow-up period. In a previous study on transition to psychosis in CHR patients, it was reported that anxiety disorders associated with decreased conversion rate to psychosis, and bipolar disorder as well as depressive disorder with increased conversion rate (10). Due to the small sample size in this study, the results could not confirm the impact of baseline anxiety and/or depressive diagnoses on predicting the transition to psychosis in non-clinical CHR individuals. However, in the current study the baseline comorbid mental disorders were found to be significantly associated with increased scores of three subscales of positive symptoms addressing Unusual Thought Content/Delusional Ideas, Suspiciousness/Persecutory Ideas, and Disorganized Communication at 6-month follow-up, and with increased score of one subscale of negative symptoms addressing Avolition at 6-month follow-up. These findings suggest that the comorbid mental disorders are unfavorable factors on prognosis. Previous studies reported that the prodromal stage of schizophrenia is featured as the initial signs of depressive symptoms, followed by the emergence of negative symptoms and finally the onset of psychotic symptoms (26). Copresence of psychiatric symptomatology in disorders of anxiety and depression in CHR subjects may have an accumulating impairment in psychosocial functioning (9); in addition, cooccurrence of depression contributes to poorer satisfaction with daily activities and health, and hence impaired quality of life, that decrease the resource of recovery (27). Although anxiety and depression in CHR subjects do not appear to affect conversion to psychosis, it is a prominent concern and may play a significant role on the severity of attenuated psychotic and negative symptoms. Therefore, assessment of anxiety and depression need to be considered as a significant aspect for all CHR individuals, so that they can be provided with appropriate resources and intervention to improve their prognosis.

To summarize, the strengths of the present study were the screening for CHR individuals from a large non-clinical adolescent population, the high response rates at each assessment point, the use of reliable international measures, and a longitudinal design. Our findings suggest that non-psychotic comorbid mental disorders are common presented in non-clinical youth in CHR for psychosis, and anxiety disorder is most frequent. Copresence of anxiety and/or depression is related to higher level of APS and unfavorable clinical outcome. Therefore, the routine assessment and intervention in anxiety and depression of CHR individuals are recommended, and especially such a prevention system against psychosis in general adolescent population should be established.

This study has some limitations. One limitation of this study is that information about the onset of comorbid mental disorder was not fully collected and therefore limits the capability to determine if the comorbid mental disorders preceded or followed the development of CHR criteria. Second, the sample size is small and thus it restricts the extent to explore the impact of different mental disorder on the APS, respectively. Other limitations include not having data on the course of comorbid mental disorder between baseline and end of follow-up, and only using the GAF to evaluate the general functioning for the CHR population. Further longitudinal studies with larger sample sizes are needed to investigate the characteristics of comorbid mental disorders in CHR subjects in general population, and interaction between the comorbid mental disorders and APS as well as the general functioning. In addition, it is necessary to evaluate the effectiveness of interventions focusing on reducing the impact of anxiety and depression, hence improving the prognosis in individuals at CHR.

## Ethics Statement

This study was carried out in accordance with the recommendations of Ethics Committee of the School of Medicine, Tongji University, Shanghai, China (Protocol no. 2014YXY16), with written informed consent from participants of the study.

## Author Contributions

JS contributed to study design, recruitment of participants, data analysis, interpretation of data, and writing of the manuscript. LW contributed to recruitment of participants and interpretation of results. YY contributed to recruitment of participants. NS contributed to recruitment of participants. XZ contributed to study design and interpretation of results. CZ and ZM contributed to recruitment of participants. All authors have approved the final manuscript.

## Conflict of Interest Statement

The authors declare that the research was conducted in the absence of any commercial or financial relationships that could be construed as a potential conflict of interest.
